# Molecular identification of isolates of the *Trichophyton mentagrophytes* complex

**DOI:** 10.7150/ijms.35173

**Published:** 2020-01-01

**Authors:** María Guadalupe Frías-De-León, Erick Martínez-Herrera, Carlos Enrique Atoche-Diéguez, José Luís González- Cespón, Brianda Uribe, Roberto Arenas, Carmen Rodríguez-Cerdeira

**Affiliations:** 1 Research Unit, High Speciality Regional Hospital of Ixtapaluca. Ixtapaluca, Edo. Mexico.; 2 Dermatological Center of the Southeast “Dr. Fernando Latapí”, Mérida, Yucatán, México.; 3 Efficiency, quality and costs in Health Services Research Group (EFISALUD), Galicia Sur Health Research Institute (IIS Galicia Sur). SERGAS-UVIGO.; 4 Mycoloy Service, Hospital Manuel Gea González, Mexico City, Mexico.; 5 Dermatology Department, Hospital do Meixoeiro and University of Vigo, Vigo, Spain.; 6 European Women's Dermatologic and Venereologic Society (EWDVS), Tui, Spain.; 7 Psychodermatology task force of the Ibero-Latin American College of Dermatology (CILAD).

**Keywords:** *Trichophyton mentagrophytes complex*, * Trichophyton mentagrophytes*, *Trichophyton interdigitale*, Sabouraud dextrose agar, Internal transcribed spacer

## Abstract

**Background:** The *Trichophyton mentagrophytes* complex is the second most common causal agent of dermatophytosis. It comprises five species—*T. mentagrophytes*, *T. interdigitale*, *T. erinacei*, *T quinckeanum,* and* T. benhamie,* as well as nine different genotypes of* T. mentagrophytes* / *T. interdigitale*—which are morphologically similar; however, their susceptibility to antifungal agents may differ. For targeted therapy and better prognosis, it is important to identify these species at a molecular level. However, since many hospitals lack molecular methods, the actual aetiology of dermatophytosis caused by this complex remains unknown.

**Objective:** To characterize 55 anthropophilic isolates of the *T. mentagrophytes* complex recovered from a dermatological centre in Yucatán, Mexico.

**Material and methods:** Fifty-five isolates of the *T. mentagrophytes* complex were obtained from patients with *tinea capitis*, *tinea pedis*, *tinea corporis*, *tinea barbae*, and *tinea unguium*. They were characterized by their colonial and microscopic morphology on Sabouraud dextrose agar (SDA) and through the sequencing of a fragment from the region ITS1-5.8S-ITS2.

**Results:** All colonies grown on SDA were white. Forty-six isolates formed colonies with a powdery texture, while nine isolates formed colonies with a velvety texture. The micromorphological features were typical of the *T. mentagrophytes* complex. The molecular analysis revealed that 55 isolates were microorganisms that belonged to the *T. mentagrophytes* complex, that 46 formed powdery colonies representing *T. mentagrophytes*, and that the other nine isolates that formed velvety colonies represented *T. interdigitale*. The latter nine isolates were obtained from patients with *tinea pedis*, *tinea corporis*, and *tinea unguium*.

**Conclusions:** The colony morphology on SDA led to the identification of 46 isolates as *T. mentagrophytes* and nine isolates as *T. interdigitale*. At a molecular level, the species identified by their morphology were identified only as *T. mentagrophytes* complex.

## Introduction

Dermatophytes are a group of fungi that are closely related to each other and have the enzyme keratinase; thus, they can cause infections in the skin, hair, and nails in both humans and animals^1^. Among the dermatophytes, *Trichophyton mentagrophytes* stands out as the second most common causative agent of dermatophytosis^2^,^3^, after *T. rubrum*. This fungus is characterized morphologically based on the development of macro and microconidia with smooth walls. The macroconidia originate laterally in the hyphae or in short pedicles of thin or thick walls and are club-shaped or fusiform, with a size that varies from 4-8 to 8-50 μm. The microconidia are abundant, spherical, pyriform, or irregularly shaped, with sizes varying from 2-3 to 2-4 μm. The most consistent feature of *T. mentagrophytes* is the production of globose micro-aleuriospores arranged in groups (like a bunch of grapes)^3^. The taxonomy of *T. mentagrophytes* is complex due to the changes it has undergone in recent years. Until 2017, *T. mentagrophytes*-series included seven species: *T. tonsurans*, *T. mentagrophytes*, *T. interdigitale*, *T, equinum*, *T. quinckeanum, T. schoenleinii*, and* T. simii* characterized through ecological data, morphological characteristics, mating type studies, and molecular analysis^4^.

However, nowadays, only five species are considered—*T. mentagrophytes*, *T. interdigitale*, *T. erinacei*, *T quinckeanum*, and* T. benhamie*—as well as nine different genotypes of *T. mentagrophytes* / *T. interdigitale* associated with the geographical origin and the source of infection^5^. These species differ with regards to their ecological preferences; for example, *T. interdigitale* is anthropophilic and produces dispersal aerial mycelium with numerous conidia, while *T. mentagrophytes* is zoophilic and produces powdery colonies^6^. Conventionally, *T. mentagrophytes (sensu lato)* is identified based on its macro and microscopic features, and sometimes, for its physiological characteristics (hair perforation and urease activity), particularly in the case of atypical isolates^7^,^8^; however, the results are usually uncertain due to phenotypic variations among isolates, such as the mycelial growth rate, the colour (white or beige) and appearance of colonies (powdery or velvety), the number of microconidia, the presence or absence of spiral filaments, etc^9^. In clinical practice, it is common for the anthropophilic velvety isolates obtained from the human foot to be named as *T. interdigitale*, while the powdery isolates, regardless of their origin, are categorised as *T. mentagrophytes*^9^. However, this strategy is insufficient and unsuitable to differentiate *T. mentagrophytes* from the rest of the species that can cause infection in humans; additionally, like *T. interdigitale*, it also produces velvety colonies. Therefore, to ensure the accuracy of the identification, the use of molecular methods is recommended in combination with morphological analyses^2^,^6^,^10^,^11^.

The treatment used for *tinea* caused by *T. mentagrophytes sensu lato* is, in general, effective against all members of the complex; however, an increase in the number of cases caused by *T. interdigitale* resistant to terbinafine, which is the treatment of choice, has been reported^12^-^14^. Thus, in order to achieve a targeted therapy and better prognosis, and for epidemiological purposes, it is important to perform the identification of the fungus at a species level using molecular methods^11^.

In Mexico, many hospitals lack the infrastructure to identify the species of the *T. mentagrophytes* complex at a molecular level. Thus, conventional methods are used habitually, which leads to a lack of awareness of the actual aetiology of the dermatophytosis.

The present study aimed to characterize the anthropophilic isolates of the *T. mentagrophytes* complex recovered from a dermatological centre in the Yucatán Peninsula, Mexico, through the conventional method (cultivation on Sabouraud dextrose agar (SDA)) and the molecular method (sequencing of a fragment of the internal transcribed spacer (ITS) region).

## Materials and methods

### Isolates

In total, 55 patients (22 men and 33 women) with an age range of 1-80 years, a clinical suspicion of infection with the* T. mentagrophytes* complex, and a clinical presentation of* tinea capitis*, *tinea corporis*, *tinea unguium*, *tinea pedis*, and *tinea barbae* that were referred to the Central Mycology Laboratory of Dermatological Center "Dr. Fernando Latapí" in Mérida, Yucatán, Mexico, from 2006 to 2016, were studied for 11 years. The fungi had been identified through the morphological characteristics observed via direct examination with lactophenol cotton blue and were preserved in tubes with SDA (Bioxón, CDMX, MX) and actidione at 4°C.

### Morphological characterization

The isolates were inoculated on SDA and were incubated at 28°C for 7 days. The macroscopic and microscopic characteristics of the colonies were observed through microcultures^15^.

### Molecular characterization

#### DNA Extraction

From the culture of each isolate on SDA plates, a block of approximately 1 cm^3^ was cut out and transferred into flasks that contained Malt Extract Broth (Bioxon), followed by incubation at 25°C under conditions of agitation for 7-10 days. The mycelium of each isolate was filtered, lyophilized, and preserved at 4°C. The total fungal DNA was extracted from the mycelium using the Animal and Fungi DNA Preparation Kit (Jena Bioscience Gmbh, Jena, TH, DE), following the manufacturer's instructions. The DNA obtained was analysed using a Nanodrop (Thermo Scientific, Waltham, MA, USA) at 260 nm.

#### PCR and sequencing

The amplification reactions of a fragment of the ITS1-5.8S-ITS2 region were carried out using the oligonucleotides ITS1 (5′-tccgtaggtgaacctgcgg-3′) and ITS4 (5′-tcctccgcttattgatatgc-3′) (Sigma Aldrich Co Ltd., Missouri, USA), which produced a 600 to 750-bp-long fragment, according to the process described by Ziółkowska et al.^16^. The amplification products were analysed by electrophoresis on a 1.5% agarose gel, followed by staining with GelRed™ 3x (Biotium, Fremont, CA, USA) in TAE buffer (Tris-Acetate-EDTA) 1X. A 100-bp DNA ladder (Fermentas Life Sciences, Waltham, MA, USA) was used as the molecular-weight marker. The images from the gels were visualized and documented with the Gel Doc™ EZ Gel Documentation System (Bio-Rad Laboratories Inc., Hercules, CA, USA).

The amplicons were purified with the PCR Purification Kit (Jena Bioscience GmbH) and sequenced in both directions (Langebio, Guanajuato, Mexico). The final determination of the species was based on the comparison of the sequences of the isolates with the reference sequences (MH865908.1, MH864960.1, MH859073.1, MH865946.1, MH865915.1, and MH859166.1) in the GenBank database, using the BLAST algorithm (https://blast.ncbi.nlm.nih.gov/Blast)^17^.

To determine the phylogenetic position of the isolates of the *T. mentagrophytes complex,* a Maximum Likelihood tree was built. This analysis was carried out using RAxML v.8.0.0 software. To obtain 1000 bootstrap replicates, the tree-bisection-reconnection method was used and the GTR+G evolutionary model obtained with the JModeltest 2.1.10 program using the Bayesian Information Criterion (BIC) was employed.

## Results

**Figure 1:**
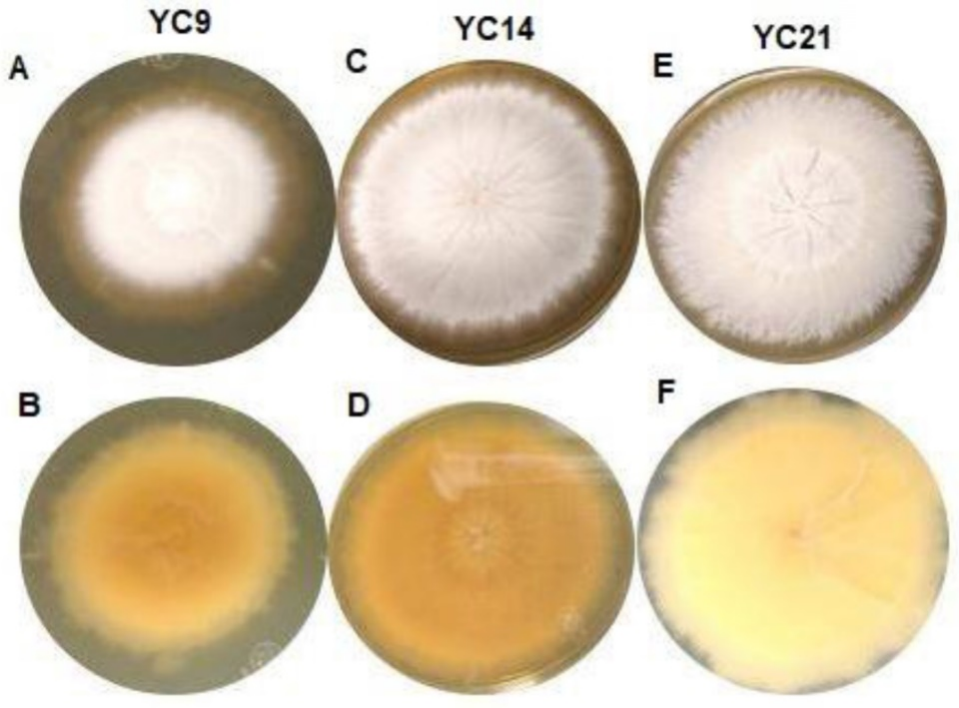
Macroscopic view of the isolates from the *Trichophyton mentagrophytes* complex cultured for 10 to 15 days on Sabouraud dextrose agar at 25-30°C. A: Isolate YC9, obverse view showing white-coloured, limited-growth colony with velvety texture. B: Isolate YC9, reverse view showing yellow-coloured colony with no diffusible pigment. C: Isolate YC14, obverse view showing white-coloured colony with radial, unlimited growth and powdery texture. D: Isolate YC14, reverse view showing yellow-coloured colony with no diffusible pigment. E: Isolate YC21, obverse view showing white-coloured colony with unlimited growth and granular texture. F) Isolate YC14, reverse view showing beige-coloured colony with no diffusible pigment.

All the studied isolates formed white colonies on SDA; however, 46 isolates formed powdery colonies with a brown-yellowish pigmentation on the back, while nine isolates formed colonies with a velvety surface and yellowish pigmentation on the reverse (Figure 1), suggestive of *T. mentagrophytes* and *T. interdigitale*, respectively.

**Figure 2:**
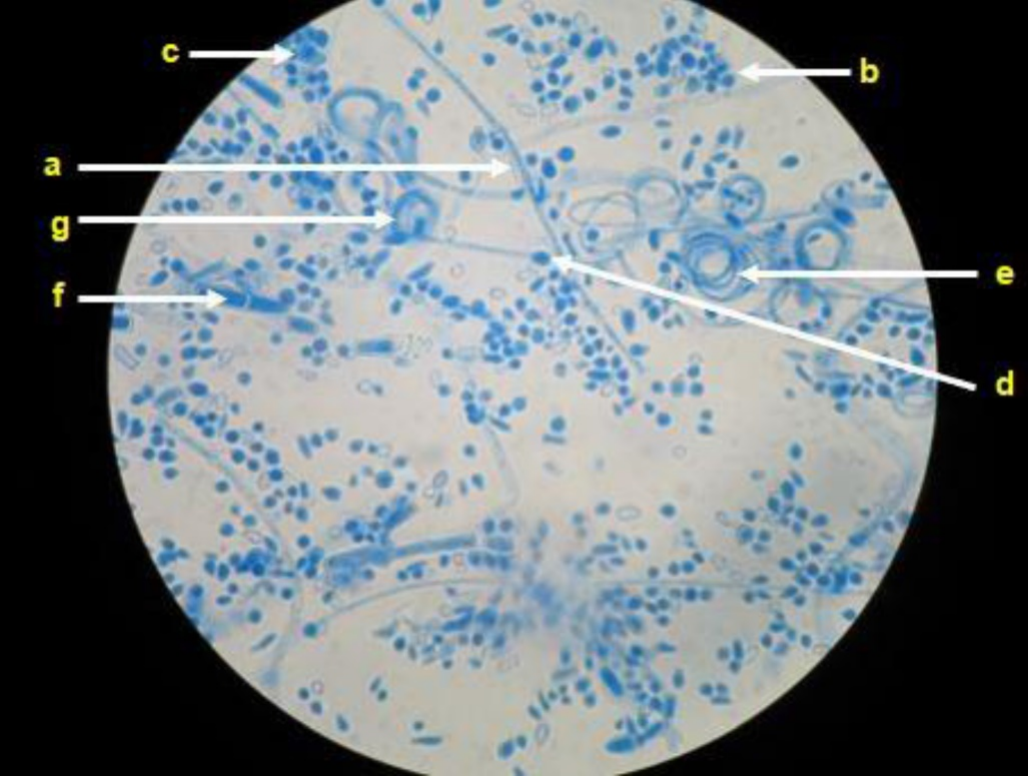
Representative microscopy image. Hyaline, septate, and branched hyphae (a) can be observed in *Trichophyton mentagrophytes* complex, as well as abundant spherical or semi-spherical microconidia (b) that resemble clusters of grapes (c), spherical chlamyconidia (d), spiral hyphae (e), macroconidia (f) and nodular bodies (g).

Upon the micromorphological analysis of the 55 isolates, the presence of branched and septate hyaline hyphae was observed; in case of eight isolates, it was possible to observe the formation of spiral hyphae, particularly in fungi that developed velvety colonies. Spherical and/or pyriform microconidia were found in case of all the isolates but were abundant in those that formed powdery colonies, where they were observed to be alternating through the length of the hyphae (Cross of Lorraine). Additionally, club-shaped, multiseptate macroconidia were visualized in case of 20 fungal isolates that formed powdery colonies (Figure 2).

**Figure 3:**
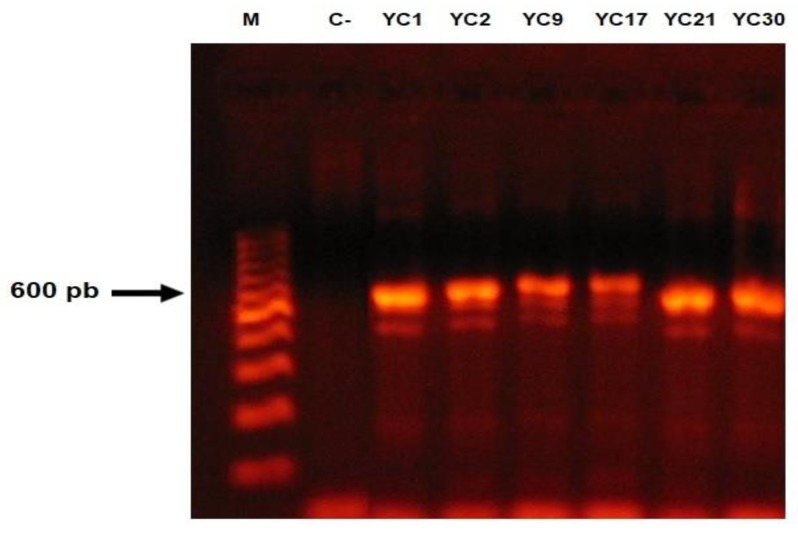
Amplification reactions of a fragment of the ITS1-5.8S-ITS2 region, from the fungal isolates of the *Trichophyton mentagrophytes* complex. Molecular marker (M), negative control (C-).

For all isolates, a fragment approximately 600- to 750-bp long was amplified (Figure 3). High similarity (100% identity and 100% coverage) was found by BLASTn among the sequences of the 46 isolates studied, when compared with the sequences of the *T. mentagrophytes* strains CBS (gbǀMH866908.1, gbǀMH864960.1 and gbǀMH859073.1) deposited in the GenBank database, while sequences of the nine isolates identified as the *T. interdigitale* by morphological features showed a high similarity with *T. interdigitale* strains CBS (gbǀMH865946.1, gbǀMH865915.1 and gbǀMH859166.1) deposited in the GenBank database. Importantly, the 46 sequences of *T. mentagrophytes* showed 98% identity with *T. interdigitale* and the nine sequences of *T. interdigitale* showed 99% identity with *T. mentagrophytes* strains reported in GenBank*.* The sequences of the 55 studied isolates were deposited in the GenBank database (Access No.: MK045530-MK045584). No isolates of *T. erinacei*, *T quinckeanum* or* T. benhamie* were found (Table 1).

To determine the phylogenetic position of the isolates of the *T. mentagrophytes complex,* a Maximum Likelihood tree was built, using *Nannizzia gypsea* (Accession Number KT155807.1) as an outgroup. The phylogenetic tree formed four groups. Group I included the reference sequence for *Trichophyton erinacei* (Accession Number KT155878.1). Group II included the reference sequence *Trichophyton quinckeanum* (Accession Number KY680503.1), with a 86% bootstrap. Group III included the reference sequence for *Athroderma benhamiae* (Accession Number AF506034.1) and *Trichophyton mentagrophytes* (Accession Number KT155726.1) with an 84% bootstrap. Finally, group IV was composed of 55 isolates from the study that were associated with the reference sequence of *Trichophyton interdigitale* (Accession Number KC595991.1) with an 85% bootstrap.

## Discussion

In clinical practice, the name *T. mentagrophytes* is still used incorrectly to name dermatophytes that form powdery or cottony-white colonies with yellowish pigmentation on the back, and show multiseptate macroconidia, pyriform microconidia grouped in clusters, and spiral hyphae^3^. This error occurs for two reasons: first, dermatologists do not consider the possibility that all species comprising the *T. mentagrophytes* complex, including zoophilic fungi, can cause infection in humans^6^, and second, the identification of *T. mentagrophytes* is performed through the correlation of the clinical manifestations of the infection with a microscopic examination of the morphology, and eventually, in combination with physiological tests^10^, since these methods do not allow the differentiation between the four species of the *T. mentagrophytes* complex.

The microscopic characteristics of these species are practically identical, while macroscopically, their growths on SDA have been reported to be indicative of the identification of the most frequent species, particularly, *T. mentagrophytes* and *T. interdigitale*^18^,^19^. However, the variability that the fungus can present in its morphologic features with subcultures, prevents these characteristics from being considered as specific markers of each species; thus, the use of more specific methods, such as molecular techniques, are necessary for their proper identification^11^. In this study, the macromorphological findings pertaining to the growth of the fungi on SDA were oriented towards the presence of two types of fungal isolates, since colonies with two types of textures, i.e. powdery and velvety, were observed, which have been associated with *T. mentagrophytes* and *T. interdigitale*, respectively^18^. Additionally, although all the isolates of the complex present the typical micromorphology of *T. mentagrophytes sensu lato*, different characteristics among the species were observed: the presence of macroconidia and/or abundance of microconidia was observed in 100% of the *T. mentagrophytes* isolates, while in 88.9% of the *T. interdigitale* isolates, the presence of spiral hyphae^20^ stood out.

Concerning the molecular data, the Blastn analysis showed that the sequences of the STIS1-5.8S-ITS2 marker of *T. mentagrophytes* and *T. interdigitale* are very similar. This echoes the data of de Hoog et al.^4^, who were only able to identify groups at the genus level with this marker. The same situation occurred in the present study, and because *T. mentagrophytes* and *T. interdigitale* are very close, they were included within the same group in the phylogenetic tree. Therefore, from a multilocus phylogeny, Hoog et al.^4^ proposed better-resolved clades in which the *T mentagrophytes* series falls within the clade A-1 that includes seven species (*T. mentagrophytes*, *T. interdigitale*, *T. simii*, *T. tonsurans*, *T. equinum*, *T. quinckeanum* and *Trichophyton schoenleinii)*^4^*.* Considering that in this study the sequencing was only performed with the ITS marker, it was confirmed at the molecular level that the morphological identification of the fungi was the genus *Trichophyton*.

Nennoff et al.^5^ proposed a new classification for the *T. mentagrophytes* complex according to the data reported by Heidemann et al.^21^ and the results obtained by them in a study performed with population from India using the molecular marker ITS, in which they assure that this marker is good for the differentiation between the species *T. mentagrophytes and T. interdigitales.* The authors proposed nine genotypes based on the phylogenetic comparison of different sequences obtained from these two species of fungus isolated from different regions of the world. In the present study, the problematic sequences were evaluated by comparing them with the sequences used by Nennoff et al^2^. They all grouped in the same branch without distinction. Thus, it may be considered that the ITS marker is not resolute and that these fungi may belong to ITS type I, ITS Type III or both.

However, these results do not justify the use of the culture on SDA for a presumptive identification of these species*,* since the five species of the *T. mentagrophytes* complex can cause infection in humans^8^,^18^,^22^,^23^; however, some fungi, such as *T. erinacei*, are rare6. Furthermore, some fungi may be resistant to first-line antifungal drugs^14^. The isolates identified as *T. interdigitale* were obtained from two patients with *tinea pedis*, five with *tinea corporis*, and two with *tinea unguium*, which coincides with findings of other studies, in which this fungus has increasingly been identified as the causal agent of *tinea* with different clinical manifestations^8^,^11^,^16^,^18^. Conducting molecular studies on a regular basis is recommended to gain a better understanding of the epidemiology of the infections caused by the *T. mentagrophytes* complex.

## Conclusions

In conclusion, considering the difficulties associated with the identification of dermatophytes and the relatedness of the taxa in our study group, the amplification reactions of a fragment of the ITS1-5.8S-ITS2 region did not provide a powerful alternative for the identification of *T. interdigitale*. At the molecular level, the species could only be identified as *T. mentagrophytes* complex. Since the morphological analysis of colonies in SDA led to their preliminary identification, it should not be ruled out for the identification of species in this complex.

Therefore, it is necessary to use a polyphasic approach of classification, using the ITS marker along with other markers or genes during the molecular analysis, to identify the *T. mentagrophytes* complex at a species level.

## Figures and Tables

**Figure 4:**
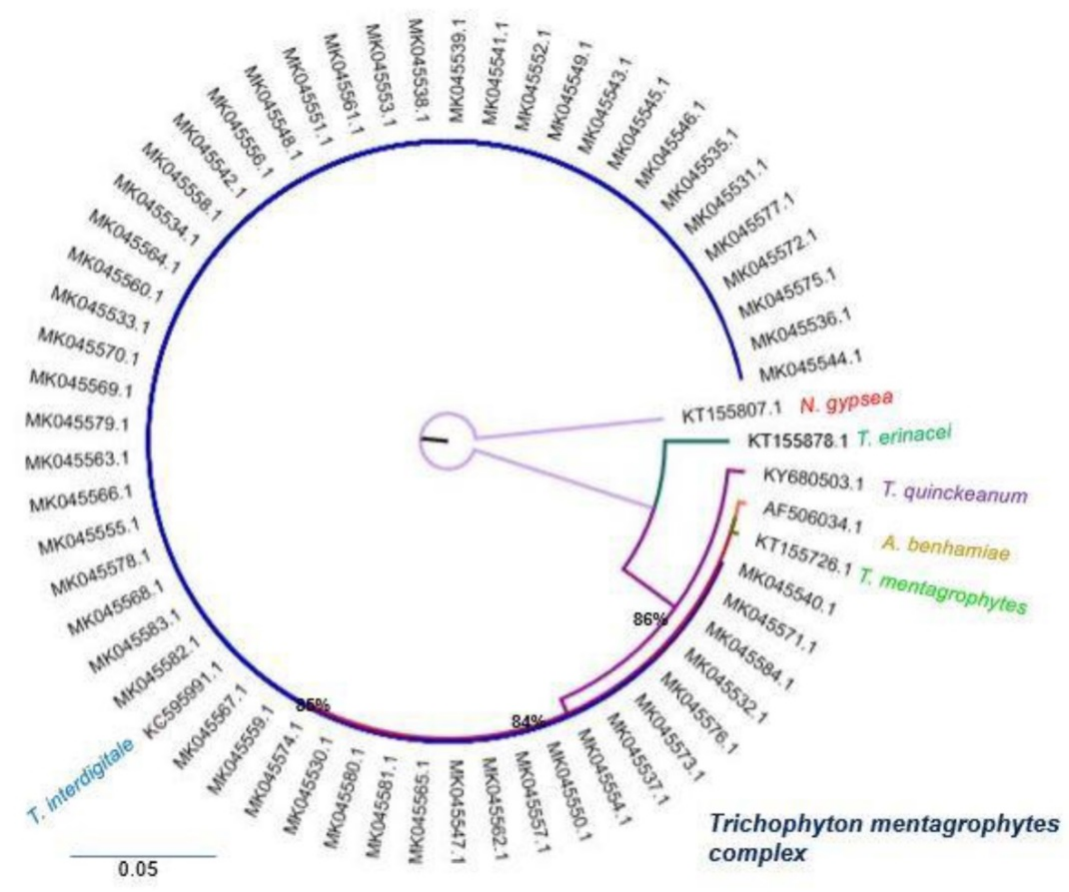
Maximum likelihood Phylogenetic tree internal transcribed sequence rDNA, (RAxML v.8.0.0), sequences of *Trichophyton interdigitale* and *Trichophyton mentagrophytes* using GTR+G as model substitution, with 1000 bootstrap replications, shown when >70%, *Nannizzia gypsea* (Accession Number KT155807.1) was selected as outgroup and as reference strains *Trichophyton mentagrophytes* (Accession Number KT155726.1), *Trichophyton interdigitale* (Accession Number KC595991.1), *Trichophyton quinckeanum* (Accession Number KY680503.1), *Trichophyton erinacei* (Accession Number KT155878.1) and *Athroderma benhamiae* (Accession Number AF506034.1).

**Table 1:** Conventional and molecular characterization of the anthropophilic isolates of the *Trichophyton mentagrophytes* complex examined in this study

Isolate	Initial identification/molecular characterization species	Accession number	Sex/Age (years)	Area	Source/Conventional characterization	Colonies on SDA
YC1	*T. mentagrophytes/T. mentagrophytes*	MK045530	Male/8	Urban	Hairy skin / *tinea capitis*	Granular
YC2	*T. mentagrophytes/T. mentagrophytes*	MK045531	Female/10	Rural	Hairy skin / *tinea capitis*	Granular
YC3	*T. mentagrophytes/T. mentagrophytes*	MK045532	Male/3	Urban	Hairy skin / *tinea capitis*	Granular
YC4	*T. mentagrophytes/T. mentagrophytes*	MK045533	Male/7	Rural	Hairy skin / *tinea capitis*	Powdery
YC5	*T. mentagrophytes/T. mentagrophytes*	MK045534	Female/4	Rural	Hairy skin / *tinea capitis*	Granular
YC6	*T. mentagrophytes/T. mentagrophytes*	MK045535	Female/13	Rural	Hairy skin / *tinea capitis*	Granular
YC7	*T. mentagrophytes/T. mentagrophytes*	MK045536	Female/12	Rural	Hairy skin / *tinea capitis*	Granular
YC8	*T. mentagrophytes/T. mentagrophytes*	MK045537	Female/4	Urban	Hairy skin / *tinea capitis*	Granular
YC9	*T. mentagrophytes/T. interdigitale*	MK045538	Female/48	Rural	Nail / *tinea unguium*	Velvety
YC10	*T. mentagrophytes/T. interdigitale*	MK045539	Male/62	Rural	Nail / *tinea unguium*	Velvety
YC11	*T. interdigitale/T. interdigitale*	MK045540	Female/69	Rural	Skin /*tinea pedis*	Velvety
YC12	*T. mentagrophytes/T. interdigitale*	MK045541	Female/54	Urban	Skin /*tinea pedis*	Velvety
YC13	*T. mentagrophytes/T. mentagrophytes*	MK045542	Male/3	Rural	Hairy skin / *tinea capitis*	Granular
YC14	*T. mentagrophytes/T. mentagrophytes*	MK045543	Male/5	Rural	Hairy skin / *tinea capitis*	Powdery
YC15	*T. mentagrophytes/T. interdigitale*	MK045544	Male/38	Urban	Hairless skin / *tinea corporis*	Velvety
YC16	*T. mentagrophytes/T. mentagrophytes*	MK045545	Female/4	Urban	Hairy skin / *tinea capitis*	Granular
YC17	*T. mentagrophytes/T. mentagrophytes*	MK045546	Male/9	Rural	Hairy skin / *tinea capitis*	Granular
YC18	*T. mentagrophytes/T. mentagrophytes*	MK045547	Male/8	Rural	Hairy skin / *tinea capitis*	Granular
YC19	*T. mentagrophytes/T. mentagrophytes*	MK045548	Female/2	Rural	Hairy skin / *tinea capitis*	Granular
YC20	*T. mentagrophytes/T. interdigitale*	MK045549	Male/33	Rural	Hairless skin / *tinea corporis*	Velvety
YC21	*T. mentagrophytes/T. mentagrophytes*	MK045550	Female/5	Urban	Hairy skin / *tinea capitis*	Granular
YC22	*T. mentagrophytes/T. interdigitale*	MK045551	Female/11	Rural	Hairless skin / *tinea corporis*	Velvety
YC23	*T. mentagrophytes/T. interdigitale*	MK045552	Female/80	Urban	Hairless skin / *tinea corporis*	Velvety
YC24	*T. mentagrophytes/T. interdigitale*	MK045553	Female/51	Urban	Hairless skin / *tinea corporis*	Velvety
YC25	*T. mentagrophytes/T. mentagrophytes*	MK045554	Male/6	Urban	Hairy skin / *tinea capitis*	Granular
YC26	*T. mentagrophytes/T. mentagrophytes*	MK045555	Male/2	Rural	Hairy skin / *tinea capitis*	Granular
YC27	*T. mentagrophytes/T. mentagrophytes*	MK045556	Female/10	Urban	Hairy skin / *tinea capitis*	Granular
YC28	*T. mentagrophytes/T. mentagrophytes*	MK045557	Male/5	Rural	Hairy skin / *tinea capitis*	Powdery
YC29	*T. mentagrophytes/T. mentagrophytes*	MK045558	Female/8	Rural	Hairy skin / *tinea capitis*	Granular
YC30	*T. mentagrophytes/T. mentagrophytes*	MK045559	Female/4	Rural	Hairy skin / *tinea capitis*	Granular
YC31	*T. mentagrophytes/T. mentagrophytes*	MK045560	Female/4	Rural	Hairy skin / *tinea capitis*	Granular
YC32	*T. mentagrophytes/T. mentagrophytes*	MK045561	Female/1	Urban	Hairy skin / *tinea capitis*	Granular
YC33	*T. mentagrophytes/T. mentagrophytes*	MK045562	Female/11	Rural	Hairy skin / *tinea capitis*	Granular
YC34	*T. mentagrophytes/T. mentagrophytes*	MK045563	Female/9	Urban	Hairy skin / *tinea capitis*	Granular
YC35	*T. mentagrophytes/T. mentagrophytes*	MK045564	Female/4	Rural	Hairy skin / *tinea capitis*	Granular
YC36	*T. mentagrophytes/T. mentagrophytes*	MK045565	Male/5	Rural	Hairy skin / *tinea capitis*	Granular
YC37	*T. mentagrophytes/T. mentagrophytes*	MK045566	Male/7	Urban	Hairy skin / *tinea capitis*	Granular
YC38	*T. mentagrophytes/T. mentagrophytes*	MK045567	Female/5	Rural	Hairy skin / *tinea capitis*	Granular
YC39	*T. mentagrophytes/T. mentagrophytes*	MK045568	Female/6	Rural	Hairy skin / *tinea capitis*	Granular
YC40	*T. mentagrophytes/T. mentagrophytes*	MK045569	Male/3	Rural	Hairy skin / *tinea capitis*	Granular
YC41	*T. mentagrophytes/T. mentagrophytes*	MK045570	Male/62	Rural	Hairless skin / *tinea corporis*	Granular
YC42	*T. mentagrophytes/T. mentagrophytes*	MK045571	Female/37	Urban	Hairless skin / *tinea corporis*	Granular
YC43	*T. mentagrophytes/T. mentagrophytes*	MK045572	Female/16	Rural	Hairless skin / *tinea corporis*	Granular
YC44	*T. mentagrophytes/T. mentagrophytes*	MK045573	Female/32	Urban	Hairless skin / *tinea corporis*	Granular
YC45	*T. mentagrophytes/T. mentagrophytes*	MK045574	Female/10	Rural	Hairless skin / *tinea corporis*	Granular
YC46	*T. mentagrophytes/T. mentagrophytes*	MK045575	Female/9	Urban	Hairless skin / *tinea corporis*	Powdery
YC47	*T. mentagrophytes/T. mentagrophytes*	MK045576	Female/35	Rural	Hairless skin / *tinea corporis*	Granular
YC48	*T. mentagrophytes/T. mentagrophytes*	MK045577	Male/24	Urban	Hairless skin / *tinea corporis*	Granular
YC49	*T. mentagrophytes/T. mentagrophytes*	MK045578	Female/40	Rural	Hairless skin / *tinea corporis*	Granular
YC50	*T. mentagrophytes/T. mentagrophytes*	MK045579	Female/38	Rural	Hairless skin / *tinea corporis*	Granular
YC51	*T. mentagrophytes/T. mentagrophytes*	MK045580	Male/74	Urban	Hairless skin / *tinea corporis*	Granular
YC52	*T. mentagrophytes/T. mentagrophytes*	MK045581	Female/39	Urban	Hairless skin / *tinea corporis*	Granular
YC53	*T. mentagrophytes/T. mentagrophytes*	MK045582	Male/64	Urban	Hairless skin / *tinea corporis*	Granular
YC54	*T. mentagrophytes/T. mentagrophytes*	MK045583	Male/63	Urban	Nail / *tinea unguium*	Powdery
YC55	*T. mentagrophytes/T. mentagrophytes*	MK045584	Male/40	Rural	Hairy skin / *tinea barbae*	Granular
